# The association between attention-deficit/hyperactivity disorder and early-onset alcohol dependence: A retrospective study

**DOI:** 10.4103/0019-5545.44748

**Published:** 2008

**Authors:** Sujaya Kumara R. Sringeri, Ravi Philip Rajkumar, Kesavan Muralidharan, Channapatna R. Chandrashekar, Vivek Benegal

**Affiliations:** Mental Health Unit, Liverpool Hospital, Liverpool NSW 2170, Australia, India; 1 Department of Psychiatry, National Institute of Mental Health and Neurosciences (NIMHANS), Bangalore-560 029, India

**Keywords:** ADHD, alcohol dependence, early-onset

## Abstract

**Background:**

Early onset (EO) alcohol dependence (AD) has been found to represent a subtype of alcoholism with a distinct profile and prognosis compared to late onset (LO) alcohol dependence. Externalizing disorders, especially attention deficit hyperactivity disorder (ADHD) that may continue as attention deficit hyperactivity disorder, residual type (ADD, RT) in adulthood, may increase susceptibility to early-onset AD.

**Aims:**

To examine the relationship between ADHD and ADD, RT symptoms and age at onset of AD in a sample of Indian male patients. 70 male subjects with AD presenting to the De-Addiction Services of the National Institute of Mental Health and Neurosciences (NIMHANS), Bangalore, were studied. The study had a retrospective design.

**Materials and Methods:**

Patients were examined for evidence of past ADHD in childhood and current ADD, RT using structured instruments. Chi-square tests and odds ratios were used to express the relative risk of association of ADHD with early- and late-onset AD.

**Results:**

Significantly more EO alcoholics (19/30, 63.3%) had a history of ADHD in childhood compared to LO alcoholics (7/28, 25%, *P* < 0.05) ADD, RT was also over-represented in EO probands.

**Conclusions:**

The results of this study are consistent with previous research that shows a high incidence of ADHD in early-onset alcoholics. This may have important management implications.

## INTRODUCTION

Early onset (EO) of alcohol dependence (AD) represents a discrete form of alcoholism[1,2] which is significantly associated with greater severity of alcohol-related problems, family history, childhood behavioral problems, craving, hostility, antisocial traits, mood disturbance, and poor social functioning compared to subjects with late onset (LO) alcohol dependence.[2–4] EO alcoholics and their first degree relatives may also be differentiated from families of LO alcoholics using a variety of putative risk markers.[5,6] EO alcoholism perhaps represents a more severe and heritable subtype,[7] which is associated with externalizing disorders such as attention deficit hyperactivity disorder (ADHD) and conduct disorder (CD).[2,8–11] An analysis of data from the Collaborative Study of the Genetics of Alcoholism (COGA) found that the mean age of onset of AD in those with comorbid disruptive behavior disorders was 14 ± 1.9 years.[12]

ADHD is one of the most studied disruptive behavior disorders in childhood. It usually first manifests by the age of 7 and is more common in boys (M: F = 2-3:1). Outcome studies of children with ADHD have showed that the disorder can persist into adolescence in 50-80% of cases.[13,16] Although overactivity mellows down into subjective restlessness, attentional problems and impulsivity continue, leading on to problems at school or work, peer relationships and low self-esteem.

Various studies have examined the co-occurrence of ADHD and substance use disorder in adolescents and adults. ADHD has been robustly associated with nicotine use disorder in mid-adolescence,[17] as well as with alcohol and other substance use disorders in late adolescence and early adulthood.[18,19] Biederman *et al*.[20] reported a prevalence of substance dependence in 21% of adults who had ADHD in childhood compared to 12% in controls. Subsequent studies also found high rates of comorbidity between ADHD and substance abuse, ranging from 24 to 47%.[10,21–23] A follow-up study[24] of children with ADHD found alcohol dependence in 16.6% of patients with mild attentional difficulties and 33.3% in those with severe attentional difficulties. The same authors followed up this cohort for 25 years, however, and found that this association was largely mediated by conduct symptoms.[25] The association between attentional impairment and substance use was corroborated by Tapert *et al*.[26] Impulsivity, which is another key feature of ADHD, has been associated with AD.[27] Milin *et al*.[28] reported a high prevalence of ADHD and ADD, residual type (RT) symptoms in patients with substance use disorder. Finally, the age of onset of AD in patients with ADHD has been found to be earlier.[29,30]

## MATERIALS AND METHODS

### Sample description

70 male subjects aged 16-60 years, admitted for alcohol related problems to the De-addiction Centre at the National Institute of Mental Health and Neurosciences, India, who met ICD-10 Diagnostic Criteria For Research[31] for Alcohol Dependence Syndrome were recruited for the study. Only those subjects whose parents (preferably mothers) reported adequate knowledge of patient's childhood, and were available for interview, were chosen. Diagnosis of AD was made clinically by obtaining information from as many informants as available. In order to minimize recall bias, the ages of onset of craving, tolerance, and withdrawal were obtained and the average of the three was taken to indicate the age of onset of dependence. The diagnosis was confirmed in each case by two independent clinicians (SK and VB). Subjects with co-morbid neuro-psychiatric illness or dependence on any other substance except nicotine were excluded.

### Procedure

The patients who satisfied the criteria for AD were assessed and divided into Early Onset (EO, those who had developed dependence before the age of 25 years) and Late Onset (LO - developed dependence after 25 years) groups. To minimize the potential overlap between these groups, subjects who had developed dependence after the age of 30 yrs were purposively selected. The severity of AD was rated using the Short Alcohol Dependence Data (SADD) questionnaire.[32]

Two or more first degree relatives who lived in close contiguity to the subject - preferably the subjects’ mother and/or older siblings - who by reason of the extended or joint family systems commonly found in India, were likely to be most informative - were then interviewed to collect information on family history of AD, childhood symptoms of ADHD and current ADD, RT. If the mother or older sibling was not available, information was collected from at least three first-degree relatives. Family history of AD was collected using the Family Interview for Genetic Studies FIGS [33] and retrospective history of ADHD was gathered using the Parent Rating Scale for ADHD (PRS). The PRS is a modification of Conner's abbreviated teachers’ rating scale, with 10 items each scored between 0 and 10. A score of 12 or more places the person above the 95^th^ percentile of childhood hyperactivity. The PRS has been used by earlier researchers[28,34,35] to retrospectively assess childhood ADHD. The presence of ADD, RT during adult life was assessed using the Wender-Utah criteria for diagnosis of ADD, RT,[36] based on information derived from the proband, his spouse and one other first-degree relative. Where a spouse was not available, another first-degree relative was interviewed. This checklist, based on a modified version of the DSM III-R criteria for ADD, RT, assesses the presence of both attentional deficits and hyperactivity and at least two other signs and symptoms such as affective lability, hot temper, and sensitivity to stress. The symptoms must occur in the absence of schizophrenia, schizoaffective disorder, primary affective disorder, schizotypal or borderline personality disorder. Data on family history, ADHD and ADD, RT were collected by an independent clinician (SK), blind to the age-at-onset status of the patient.

## RESULTS

70 subjects with AD were recruited. Of these, 37 had an EO of dependence and 33 had a LO. Of these, the records of seven subjects in the EO group and five in the LO group had to be discarded as there was insufficient data on both childhood ADHD and adult ADD, RT symptoms.

The EO subjects had a significantly lower age at onset of AD than the LO subjects (22.08 ± 3.38 years vs. 36.73 ± 6.35 years; *t* = 12.36, *P*<0.001). The two groups did not differ significantly on other demographic or clinical variables such as education, income, religion, marital status, residential status, and severity of alcoholism scores on the SADD.

Table 1 shows the prevalence of ADHD and ADD, RT in the two groups. A significantly larger number of EO alcoholics had a positive history of ADHD in childhood compared to LO alcoholics (χ^2^ = 4.3758; *P* < 0.05) and there was similar over-representation of ADD, RT in EO probands (χ^2^ = 8.9318; *P* < 0.01).

**Table 1:** Frequency of ADHD and ADD, RT in early- and late-onset alcoholics

	Early onset (%)	Late onset(%)	χ^2^ (Significance)
Childhood ADHD			
Present	19 (63.3)	7 (25)	4.3758 (P < 0.05)
Absent	7 (23.3)	10 (35.7)	
No data	4 (13.3)	11 (39.4)	
ADD, RT			
Present	17 (56.7)	5 (17.9)	8.9318 (P < 0.01)
Absent	8 (26.7)	16 (57.1)	
No data	5 (16.7)	7 (25)	

Figures in parentheses are in percentage

The odds ratio of a subject with ADHD and/or ADD, RT developing EO alcoholism was 5.8. This was in contrast to the odds ratio of 0.17 obtained for subjects with ADHD and/or ADD, RT developing LO alcoholism [Table 2].

**Table 2:** Risk of early- and late-onset alcoholism in patients with ADD and ADD, RT

Group	EO	No EO	Odds ratio
ADHD/ADD, RT	22	9	5.81
No ADHD/ADD, RT	8	19	
	**LO**	**No LO**	

ADHD/ADD, RT	9	22	0.17
No ADHD/ADD, RT	19	8	

## DISCUSSION

A significantly higher number of the EO subjects had a history of ADHD and/or ADD, RT compared to the LO subjects. The results of this study are consistent with previous research that show a high incidence of childhood ADHD and ADD, RT in substance abusers.[10,22,37–40]

The odds ratio of subjects with ADHD and/or ADD, RT developing EO dependence was nearly 6:1 compared to those without comorbid ADHD. This supports the evidence that the presence of externalizing symptoms, especially ADHD, is an important risk factor for AD at an early age, and is in line with previous studies in this patient population.[12,17] This association did not hold true for patients with LO of AD, further supporting the notion that it is the EO type of alcoholism, and not AD as a whole, that is specifically associated with childhood externalizing disorders.[2]

The prevalence of ADHD in our sample was higher than in previous studies. However, most previous studies[10,21,22] have examined the comorbidity between AD in general and ADHD, and a study which examined families with a dense pedigree[23] found a prevalence of 47%. This study on the other hand contrasted EO subjects with LO, which may account for the higher rates of ADHD found in our sample.

70.8% of all subjects (17 of 24) in our study who had evidence of ADHD in childhood also had ADD, RT as adults. This data lends support to the finding that ADHD symptoms persist from childhood into adulthood in over 50% of cases.[15,16,40]

Several studies have shed light on the mechanisms underlying the association between disruptive behavior disorders and ADHD. Kuperman *et al*.[12] found that disruptive behavior disorders precede the onset of substance dependence, and therefore represent a proximal step in the trajectory from childhood psychopathology to AD, a finding that was corroborated in a later retrospective study.[41] Various mechanisms have been proposed to explain this association, including attentional deficit,[24,26] impulsivity[27] and serotonergic dysfunction,[8,11] while others are still being elucidated.

The findings of this study must be interpreted in the context of several methodological limitations. Firstly, the retrospective assessment of ADHD relies heavily on the observation of informants, which may be coloured by recall and confounding biases, especially when delineating ADD, RT from alcohol-related behaviors. Secondly, the study sample was selected from a tertiary care center, and may have included severely ill patients, accounting for the high rates of comorbid ADHD. Other comorbidities, especially externalizing disorders, were not assessed, and the clinical profile of the two subgroups, apart from severity of dependence, was not compared, making it difficult to draw conclusions about the impact of comorbid ADHD. Thirdly, we do not have information on all those subjects who were screened and excluded from the study, or their socio-demographic or clinical variables. These people were excluded from the study at screening itself because neither parent was available to provide information. Finally, the study was cross-sectional in origin, so that course and outcome could not be commented upon.

Nevertheless, these results strongly suggest a need for greater evaluation of ADHD in populations of adults with AD, especially those with an EO of AD, and more intensive management of this high-risk group in view of their poorer prognosis. Since treatment of ADHD in adolescents, including stimulants, is known to reduce substance use, including alcohol use,[42,43] assessment of comorbid ADD, RT in adults has important therapeutic implications.
